# Cremation

**Published:** 1881-09

**Authors:** 


### Cremation.

A large meeting was recently held at Bologna to advocate the introduction of cremation instead of burial as a means of disposal of the dead of the town. Many physicians are reported to have been present, and to have taken an active interest in furthering the proposition. It seems probable that Bologna will before long be provided with a public crematory, as Milan and Lodi in the north of Italy now are.

In the cremation hall just outside the city of Gotha in Germany, which is one of the best arranged of those thus far established, fifty-two bodies, five being those of women, have, since its erection, less than three years ago, been subjected to the process; of these bodies one was sent from so great a distance as New York, and one, at least, from England.

We have not at hand the exact number of bodies which have been reduced in the furnace at Washington, Pa., but believe it to be still quite small. To overcome existing prejudices against such an innovation upon old habits and customs it is essential, not only that the scientific process of combustion should be as complete, as rapid, and as imperceptible as possible, but also that the building and its surroundings should be attractive and in keeping with a solemn ceremony. The buildings at Milan and Gotha answer these requirements, the latter place, however, has the additional advantage of being provided with a Siemens' incineration apparatus in which the process of reduction takes place in superheated air, the body not being brought at any time in direct contact with the flames. Outside the cremation hall is an open portico in which the cinerary urns are arranged. Within is a hall where the religious ceremony, when such is desired, may be performed, and where the body awaits its removal to the furnace. For this nine hours of preliminary preparation are required. The air in the receptacle where the body undergoes incineration is heated to 600° Reamur, and two hours elapse from the commencement to the completion of the process before the ashes are collected. Of these the average body of a man yields about six pounds, that of a woman about four. Only the officials and one or two of the nearest relatives are admitted to the chamber underground in which the furnace is situated. The cost of the process at Gotha is about twenty-five dollars.

At Milan the entire process may be watched through a small window; and the cost of conversion by the Gorini furnace, the one there in use, is only about one dollar; in this apparatus, however, the body is subjected to direct contact with the flames. Venini's furnace has the same disadvantage, and is besides quite complicated, but it performs its work with rapidity and thoroughness.

A furnace constructed by Dr. C. W. Siemens was referred to by Sir Henry Thompson in his article on Cremation, in the *Contemporary Review*, which converted a body weighing two hundred and twenty-seven pounds, placed in a cylindrical vessel seven feet long, by five or six in diameter, the interior being heated to 2000° F., into five pounds of ashes in fifty-five minutes.

It is evident from the progress already made that the question of the best methods of cremation will receive a pretty definite and satisfactory solution long before the sentimental considerations involved are appreciably affected by argument or example. —*Boston Medical and Surgical Journal.*

